# Increased Methyl-CpG-Binding Domain Protein 2 Promotes Cigarette Smoke-Induced Pulmonary Hypertension

**DOI:** 10.3389/fonc.2022.879793

**Published:** 2022-06-16

**Authors:** Jixing Wu, Qian Huang, Qinghai Li, Yiya Gu, Yuan Zhan, Ting Wang, Jinkun Chen, Zhilin Zeng, Yongman Lv, Jianping Zhao, Jie Xia, Jungang Xie

**Affiliations:** ^1^ Department of Respiratory and Critical Care Medicine, National Clinical Research Center of Respiratory Disease, Key Laboratory of Pulmonary Diseases of Health Ministry, Tongji Hospital, Tongji Medical College, Huazhong University of Science and Technology, Wuhan, China; ^2^ Department of Respiratory and Critical Care Medicine, Qingdao Municipal Hospital, Qingdao University, Qingdao, China; ^3^ Department of Respiratory Disease, Thoracic Disease Center, The First Affiliated Hospital, Zhejiang University School of Medicine, Hangzhou, China; ^4^ Department of Science, Western University, London, ON, Canada; ^5^ Department and Institute of Infectious Diseases, Tongji Hospital, Tongji Medical College, Huazhong University of Science and Technology, Wuhan, China; ^6^ Health Management Center, Tongji Hospital, Tongji Medical College, Huazhong University of Science and Technology, Wuhan, China

**Keywords:** pulmonary hypertension, MBD2, cigarette smoke, pulmonary artery smooth muscle cells, BMP2, pulmonary vascular remodeling

## Abstract

Pulmonary hypertension (PH) is a chronic vascular proliferative disorder. While cigarette smoke (CS) plays a vital part in PH related to chronic obstructive pulmonary disease (COPD). Methyl-CpG-Binding Domain Protein 2 (MBD2) has been linked to multiple proliferative diseases. However, the specific mechanisms of MBD2 in CS-induced PH remain to be elucidated. Herein, the differential expression of MBD2 was tested between the controls and the PH patients’ pulmonary arteries, CS-exposed rat models’ pulmonary arteries, and primary human pulmonary artery smooth muscle cells (HPASMCs) following cigarette smoke extract (CSE) stimulation. As a result, PH patients and CS-induced rats and HPASMCs showed an increase in MBD2 protein expression compared with the controls. Then, MBD2 silencing was used to investigate the function of MBD2 on CSE-induced HPASMCs’ proliferation, migration, and cell cycle progression. As a consequence, CSE could induce HPASMCs’ increased proliferation and migration, and cell cycle transition, which were suppressed by MBD2 interference. Furthermore, RNA-seq, ChIP-qPCR, and MassARRAY were conducted to find out the downstream mechanisms of MBD2 for CS-induced pulmonary vascular remodeling. Subsequently, RNA-seq revealed MBD2 might affect the transcription of BMP2 gene, which furtherly altered the expression of BMP2 protein. ChIP-qPCR demonstrated MBD2 could bind BMP2’s promotor. MassARRAY indicated that MBD2 itself could not directly affect DNA methylation. In sum, our results indicate that increased MBD2 expression promotes CS-induced pulmonary vascular remodeling. The fundamental mechanisms may be that MBD2 can bind BMP2’s promoter and downregulate its expression. Thus, MBD2 may promote the occurrence of the CS-induced PH.

## Introduction

Pulmonary hypertension (PH) is a complex vascular disease characterized as a rise in mean pulmonary arterial pressure (mPAP) of ≥ 25 mmHg at rest, which is measured by right heart catheterization (RHC) (1, 2). It is a multifactorial pathophysiological disorder caused by increased pulmonary artery pressure, resulting in chronic elevation of pulmonary arterial resistance, right ventricular (RV) hypertrophy, RV failure, and ultimately death (3–5).

Data show that the incidence of PH in chronic obstructive pulmonary disease (COPD) is high (6). PH is often present as a result of hypoxia associated with COPD. However, hypoxia can’t account for all PH related to COPD. An increasing amount of evidence suggests that cigarette smoke (CS) may directly impact on the pulmonary vasculature system (7–10). The pathogenesis of PH includes pulmonary vascular remodeling and vasoconstriction, while pulmonary vascular remodeling is the fundamental mechanism in chronic PH. CS is closely associated with pulmonary vascular remodeling (11). Multiple biological processes, such as cell proliferation, migration, and cell cycle progression of pulmonary artery smooth muscle cells (PASMCs), are associated with pulmonary vascular remodeling in PH. Among these, PASMCs proliferation is the key component of the pulmonary vascular remodeling that occurs in PH (12). Cigarette smoke extract (CSE) can directly lead to PASMCs proliferation (13), which results in CS-induced pulmonary vascular remodeling. Hence, focusing on the pathogenesis of CS-induced PASMCs proliferation is one of the objectives in the development of treatment strategies for PH related to COPD. However, the specific molecular mechanisms need to be further investigated.

DNA methylation is the most common manifestation of epigenetics (13). Compelling evidence has been linking epigenetics to PH (12, 14, 15). Smoking or environmental CS exposure significantly modifies gene methylation, which is associated with diseases like COPD (16, 17). One of the regulatory elements of DNA methylation is the methyl-CpG-binding domain protein family (the MBD protein family). The MBD2 protein is part of the MBD protein family. Among all the members, MBD2 has exhibited the highest binding affinity with methylated DNA (18, 19). As a reader in DNA methylation, MBD2 can mediate transcriptional repression or activation through directly combining methylated DNA or gathering proteins to form a suppressive compound (18, 20, 21). Furthermore, MBD2 is associated with many proliferative diseases (18, 22, 23).

As a proliferative illness, PH is closely related to many different tumor-related genes. However, limited research has been conducted on the molecular mechanisms involved in CS-induced PH. As an element with multiple functions, MBD2 may be involved in the pathogenesis of CS-induced PH. Nonetheless, the critical role of MBD2 in CS-induced PH has not been clarified yet. Thus, in this study, the role of MBD2 in CS-induced pulmonary vascular remodeling was investigated.

## Materials and Methods

### Human Tissue

Human pulmonary arteries were obtained from patients undergoing surgery for pulmonary lump at Tongji Hospital, Tongji Medical College of Huazhong University of Science and Technology, Wuhan, China. Participants were assigned into the PH group and the control group according to their medical history, pulmonary function, and echocardiography. The PH group’s clinical diagnosis was mild to moderate COPD without hypoxemia. All enrolled PH patients had smoking history. All the included participants underwent echocardiography, and the peak tricuspid regurgitation velocity (TRV) was greater than 3.0 m/s in PH patients (1, 24). The exclusion criteria were comprised of left ventricular systolic or diastolic dysfunction, coronary heart disease, other chronic lung diseases, diabetes mellitus, infections, autoimmune diseases, severe hepatic, endocrine, or renal diseases, and medical treatment, including immunosuppressive agents or corticosteroids (25–28). Prior to enrollment, written informed consent was signed, and this study was approved by the Ethics Committee of Tongji Hospital. The baseline characteristics of the PH patients and controls are shown in **Table 1**.

**Table 1:** Clinical characteristics of the PH patients and the controls.

	Controls (n=4)	PH patients (n=5)	*P*-value
**Age in years**	64.3±8.4	69.4±6.7	NS
**Gender (M/F)**	4/0	5/0	NS
**Smoking, p.y**	0	45.8±7.1	***
**BMI, kg/m^2^**	23.1±2.8	21.2±3.1	NS
**FEV1/FVC%**	82.3±3.2	63.7±5.6	***
**FEV1% predicted**	98.1±12.5	79.6±11.5	NS

p.y, pack-years; BMI, body mass index; FEV1, forced expiratory volume in one second; FVC, forced vital capacity; n, number of participants. The data were expressed as Mean ± SD, unpaired t test was used for the significance between controls and the PH patients. ***P < 0.001 vs. controls. NS, no significance.

### Primary HPASMCs Culture

HPASMCs came from the ScienCell Research Laboratories (USA). Cells were cultured in DMEM-F12 containing 10% FBS (Gibco, Australia) supplemented by 1% streptomycin and penicillin at 37°C with 5% CO2.

### Western Blot Analysis

Total proteins were obtained from HPASMCs and human pulmonary arteries. The concentration of proteins was assessed with a BCA kit (Aspen, Wuhan, China). Whole lysates were isolated by SDS-PAGE and transferred to PVDF membranes that were blocked by TBST with 5% non‐fat dry milk at room temperature for 1h. The membranes were first incubated with primary antibodies at 4°C overnight and then HRP-conjugated secondary antibodies at room temperature for 1h. The western ECL substrate (Bio-Rad, California, USA) and the ChemiDocTM-XRS+imaging system (Bio-Rad, California, USA) were used to visualize the bands. ImageJ software (NIH, Bethesda, MD) was used to measure the band intensities quantitatively. The following primary antibodies were used: against β-ACTIN (Sungene Biotech, Tianjin, China), GAPDH (Sungene Biotech, Tianjin, China), MBD2 (Abcam, Cambridge, UK), Cyclin D1 (Cyclin D1, Proteintech, Wuhan, Hubei, China), PCNA (Proteintech, Wuhan, Hubei, China), and BMP2 (Proteintech, Wuhan, Hubei, China).

### Reverse-Transcriptase Quantitative PCR Assays (RT-qPCR)

The TRIzol reagent was used to extract total RNA in accordance with the manufacturer’s instructions. The NanoDrop 2000 spectrophotometer (Thermo Scientific, USA) was used to evaluate RNA purity and quantification. Total RNA was used for the generation of cDNA through reverse transcription-PCR with a first-strand cDNA reverse transcription kit (Takara, Dalian, China). The SYBR Green PCR Master Mix (Takara, Dalian, China) and a CFX96 Real-Time PCR Detection System (Bio-Rad) were used to perform real-time PCR. The primer pairs used for amplification are shown in **Table 2**.

**Table 2:** List of primers used for RT-qPCR in HPASMCs.

Human Gene	Forward Primer (5’-3’)	Reverse Primer (5’-3’)
*ACTB*	AGAAAATCTGGCACCACACCT	GATAGCACAGCCTGGATAGCA
*MBD2*	AAGTGATCCGAAAATCTGGGC	TGCCAACTGAGGCTTGCTTC
*CCND1*	GCTGCGAAGTGGAAACCATC	CCTCCTTCTGCACACATTTGAA
*BMP2*	ACTACCAGAAACGAGTGGGAA	GCATCTGTTCTCGGAAAACCT

### RNA Interfering and Adenovirus Overexpression

Small interfering RNA (siRNA) were synthesized by Ruibo Biotechnology, Co. (Guangzhou, China). The Lipo3000 transfection reagent (Invitrogen, Carlsbad, CA) was used to transfect HPASMCs with siRNA in accordance with the instructions of the manufacturer. MBD2 siRNA (MBD2si) sequences were: 5’ to 3’ GAAAGAUGAUGCCUAGUAA dTdT, and 3’ to 5’ dTdT CUUUCUACUACGGAUCAUU. The MBD2 overexpression adenovirus vector (MBD2ad) was designed by Vigene Biosciences, Co. (China) and transfected in accordance with the manufacturer’s instructions.

### Animal Models

Adult male Sprague-Dawley (SD) rats were obtained from the Experimental Animal Center of Tongji Hospital (Wuhan, China). Animal experiments were performed in line with the Animal Care and Use guidelines of the Chinese Council on Animal Care. Rats were randomly divided into two groups, the air group and the smoking group. The smoking groups were exposed to CS generated by ten Hong Jin Long cigarettes as previously noted (13). Rats’ lung sections (CS models and controls) came from Li, Q and Wu, J et al., and the phenotype data (**Supplementary Figure 1**) was acquired as previously described (13).

### Immunofluorescence Assay

The immunofluorescence assay was performed as previously described (13, 29). Human lung sections, rats’ lung sections (13), and HPASMCs were first stained with anti-α-smooth muscle actin (αSMA, Boster, Wuhan), anti-MBD2 (Abcam), and anti-Ki67 primary antibodies (Abcam), followed by staining with Alexa Fluor 594-labeled anti-mouse/rabbit, or Alexa Fluor 488-conjugated anti-rabbit/mouse antibodies (Invitrogen, Carlsbad, CA, USA), respectively. DAPI was used for staining the cell nuclei. A Pannoramic MIDI (3Dhistech, Budapest, Hungary) was used to scan and analyze the images. Mean fluorescence intensity was assessed by ImageJ software (NIH, Bethesda, MD).

### Preparation of Cigarette Smoke Extract (CSE)

Cigarette smoke extract (CSE) was freshly prepared by bubbling the smoke coming from two research cigarettes (Code 3R4F, University of Kentucky, USA), at a frequency of 1 cigarette/5 min, into a conical tube of 50 ml which contained 20 ml of culture medium, aided by vacuum extraction. A 0.22 μm filter was used to filter the extract, which was considered to be CSE of 100% concentration (13, 30, 31).

### CCK8 Assay, Cell Cycle Assay, and EDU Assay

To evaluate the role of MBD2 in the proliferation of HPASMCs, HPASMCs were treated for 24h with MBD2si and then incubated for 24h with 2% CSE. A Cell Counting Kit-8 (Dojindo, Japan) and a cell cycle detection kit (KeyGEN BioTECH, China) were used to assess cell proliferation in accordance with instructions of the manufacturer. An ELx800 Universal Microplate Reader (Bio-Tek Instruments, Inc., Winooski, VT) and a flow cytometer (BD Bioscience, USA) were used to conduct CCK8 and cell cycle analyses, respectively. Furthermore, HPASMCs proliferation was also measured by 5-ethynyl-2′-deoxyuridine (EdU) staining (RiboBio, China). Apollo 567 was used to label EdU, and the cells were examined through a fluorescence microscope.

### Transwell Assay

To perform migration assays, membranes with 8-μm pores (Corning Costar, Cambridge, MA) in 24-well Transwell^®^ plates were used. Prior to being digested and counted, the HPASMCs were first transfected with MBD2si for 24h. Approximately 10,000 cells were transferred to the upper chamber to adhere. Next, the medium in the upper chamber was substituted with 200 μl fresh medium containing 1% FBS, while 700 μL of medium with 20% FBS with or without 2% CSE was supplemented to the lower compartment. After 24h, the cells found in the membranes of the chambers at the bottom were fixed, stained with crystal violet, and then imaged.

### RNA Sequencing (RNA-Seq)

RNAs were extracted by using the TRIzol reagent according to the manufacturer’s protocol. The Agilent 2100 Bioanalyzer (Agilent Technologies, Santa Clara, CA, USA) was used to assess RNA integrity. Then the TruSeq Stranded mRNA LT Sample Prep Kit (Illumina, San Diego, CA, USA) was applied to construct the libraries based on the manufacturer’s instructions. The transcriptome sequencing and subsequent analysis were conducted by OE Biotech Co., Ltd. (Shanghai, China). The libraries were sequenced on an Illumina HiSeq X Ten platform, and 150 bp paired-end reads and raw reads for each sample were generated. Raw reads in fastq format were first processed with Trimmomatic; subsequently, the low-quality reads were eliminated to retrieve the clean reads for each sample for further analyses. HISAT2 was used to map the clean reads to the human genome (GRCh38). Cufflinks were used to calculate the FPKM of each gene, and HTSeq-count was used to obtain the read counts of each gene. The DESeq (2012) R package was used to perform differential expression. A *P*-value < 0.05, foldchange > 2, or foldchange < 0.5 were set as the thresholds for significantly differential expression.

### MassARRAY

A QIAamp^®^ DNA Mini kit (Qiagen, Germany) was used to extract genomic DNA from HPASMCs in accordance with instructions of the manufacturer. An EpiTect Bisulfite Kit (Qiagen, Hilden, Germany) was used to perform the bisulfite conversion reaction in accordance with instructions of the manufacturer. The PCR products were incubated together with Shrimp Alkaline Phosphatase according to the manufacturer’s instructions. Following *in vitro* transcription and RNase A digestion, small RNA fragments with CpG sites were obtained for the reverse reaction. The CpG methylation status of the BMP2 promoter (from + 415 to + 894 bp) was detected by the MassARRAY platform. Epityper software Version 1.0 (Agena, San Diego, CA, USA) was used to calculate the methylation ratios of the products. The detection and analysis were conducted by OE Biotech Co., Ltd. (Shanghai, China). The following methylated primers were used: 5′ - aggaagagag AAAGGGTATTTGGTTTTAGGGTTAG - 3′ (forward) and 5′ - cagtaatacgactcactatagggagaaggct CTACAAATTCAAAAAATCCCCAAC - 3′ (reverse).

### Chromatin Immunoprecipitation Quantitative PCR Assays (ChIP-qPCR)

ChIP assay was conducted with technical support by Genecreate (Wuhan, China). The cells were crosslinked with formaldehyde and then quenched with glycine. Then, the cells were centrifugated, washed, and harvested. The cell pellets were lysed in lysis buffer and sonicated. After centrifugation, the supernatant was diluted in dilution buffer, and part of the samples were taken as the input. Then, the immunoprecipitation was done by incubating overnight with MBD2 antibody (Abcam) or the control rabbit IgG antibody. Then, wash buffer was applied. Then, the samples were eluted and the crosslinking was reversed. Finally, the DNA was purified. The immunoprecipitated chromatin was quantified by RT-qPCR. The procedures of RT-qPCR were conducted as previously described. The primer sequences used for ChIP-qPCR are shown in **Table 3**.

**Table 3:** List of primers used for ChIP-qPCR in HPASMCs for BMP2 gene.

Primer Number	Forward Primer (5’-3’)	Reverse Primer (5’-3’)
Primer 1 (TSS-236~+75)	TCCTTTGAGAGCAGGGAGTGG	GGAGCGACTAGCGCAGC
Primer 2 (TSS+71~+179)	GCTCCGCTTCCCACACC	GTGATCCACTCGGCGCTG
Primer 3 (TSS+164~+334)	GCGCCGAGTGGATCACC	GCGCTAGGGATCGGCTC
Primer 4 (TSS+316~+447)	CGGAGCCGATCCCTAGC	CGCTCTCCTAGCCCTGG
Primer 5 (TSS+434~+556)	GGGCTAGGAGAGCGAGG	TCGCCAGTTGAAAGCACC
Primer 6 (TSS+550~+668)	CTGGCGAGCGCGAATGG	CAGCCATCCTGGGCGAC
Primer 7 (TSS+653~+777)	TCGCCCAGGATGGCTGC	AGGAACCAGCGCCCGAG

TSS, transcription start site. Numbers indicate the distance from the TSS. Negative numbers indicate it’s located before the TSS, and positive numbers indicate it’s located after the TSS. The primers were designed according to the BMP2’s CpG island locations and the prediction of the promoter’s locations.

### Statistical Analysis

All data were presented as mean ± standard deviation (SD). The results were analyzed with GraphPad Prism 8 (GraphPad Software Inc, San Diego, CA, USA). Statistically significant differences were determined using the one-way analysis of variance (ANOVA) or Student’s t-test. The RNA-seq and the MassARRAY assay were assessed by paired test, while other assays were assessed by un-paired test. *P* < 0.05 was regarded as statistically significant.

## Results

### MBD2 Protein Expression Was Increased in the Pulmonary Arteries of PH Patients and CS-Exposed Rats

**Figure 1:**
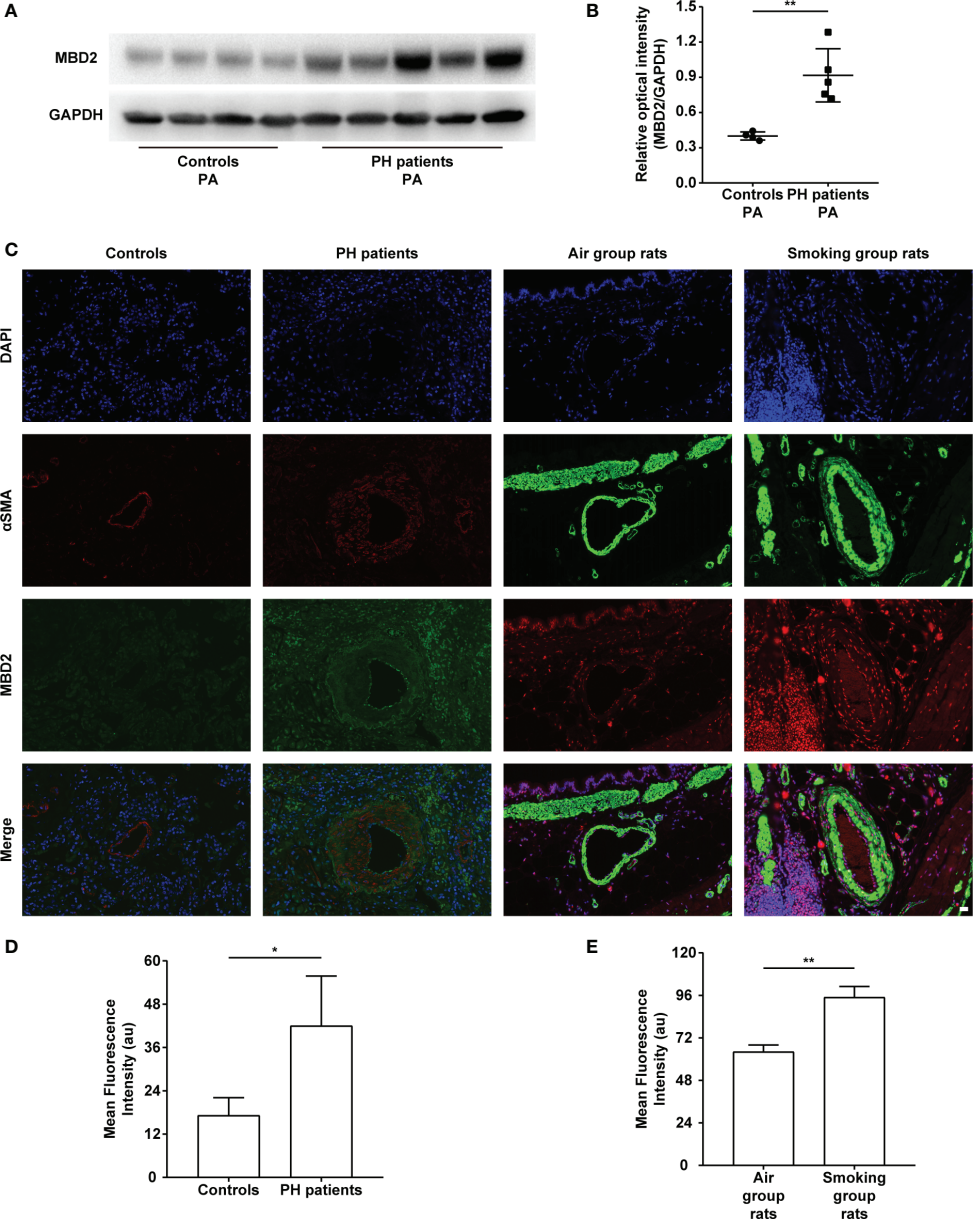
The increase in MBD2 protein in cigarette smoke (CS)-induced pulmonary hypertension (PH). **(A)** Representative Western blot images of MBD2 in human pulmonary arteries. **(B)** Quantitative analysis results of graph A based on the density of the bands (controls, n=4; PH, n=5). **(C)** Representative results of coimmunostaining of MBD2 and α-SMA in lung sections of the controls or PH groups, and the air group rats or smoking group rats. All images are 400× magnification. Scale bar = 20 μm. **(D)** Mean fluorescence intensity of the MBD2 in PAs of the controls or PH groups. (n=3) **(E)** Mean fluorescence intensity of the MBD2 in PAs of the air group rats or smoking group rats (n=3). The data are presented as mean ± SD. *P < 0.05, **P < 0.01.

The MBD2 protein expression was assessed by Western Blot. The expression of MBD2 in human pulmonary arteries of the PH groups was higher than that in the normal controls (**Figures 1A, B**). To further verify our findings, we conducted an immunofluorescence assay with tissue slices, and the MBD2 of the PH groups was confirmed to be higher than that in the controls in the pulmonary arteries of humans and CS-exposed rats (**Figures 1C-E**).

### MBD2 Protein Expression Was Elevated in HPASMCs After CSE Stimulation

**Figure 2:**
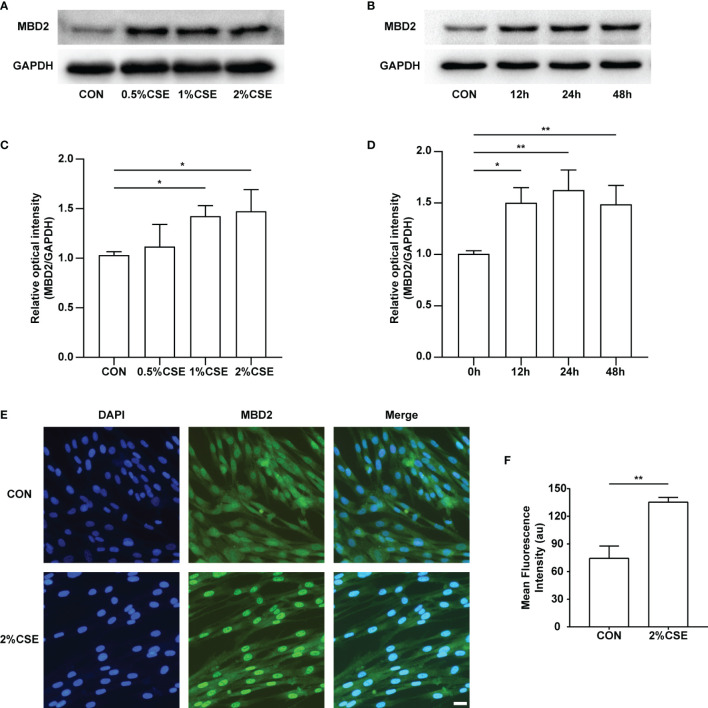
MBD2 protein increases in HPASMCs along with the concentration and duration of CSE stimulation. **(A)** Representative Western blot images of MBD2 in HPASMCs after stimulation by varying concentrations of CSE for 24h. **(B)** Representative Western blot images of MBD2 in HPASMCs after 2% CSE stimulation of different durations. **(C)** Quantitative analysis results of graph A based on the density of the bands. **(D)** Quantitative analysis results of graph B based on the density of the bands. **(E)** Representative results for immunofluorescence of MBD2 from controls and the CSE stimulation groups for 24h. All images are 200× magnification. Scale bar = 50 μm. **(F)** Mean fluorescence intensity of the MBD2 in HPASMCs. The data are presented as mean ± SD, n=3. *P < 0.05, **P < 0.01.

To examine the expression of the MBD2 protein by CSE stimulation in HPASMCs, we experimented with different concentrations (0%, 0.5%, 1%, 2%) and durations (0h, 12h, 24h, 48h) of CSE stimulation. Interestingly, we found that the expression of MBD2 was increased in the CSE stimulation groups compared with the control groups (**Figures 2A-D**, **Supplementary Figure 2**). Simultaneously, we carried out the immunofluorescence experiment to further verify the previous results. The outcomes indicated that the MBD2 protein was mainly expressed in the cell nucleus, and stimulation with CSE also elevated its expression (**Figures 2E, F**).

### MBD2si Suppressed CSE-Induced HPASMCs Proliferation and Migration

**Figure 3:**
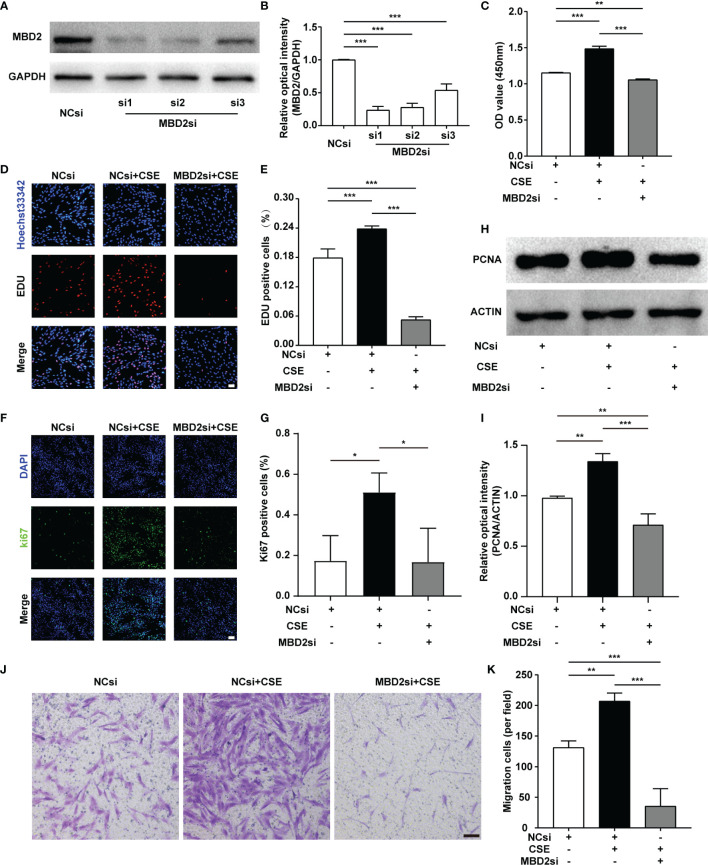
Effect of MBD2si on the changes in proliferation and migration of CSE-induced HPASMCs. **(A)** MBD2si reduced the MBD2 protein expression in HPASMCs. **(B)** Quantitative analysis results of graph A based on the density of the bands. **(C)** CCK8 test was used to assess cell proliferation in HPASMCs transfected with negative control siRNA (NCsi) or MBD2si, stimulated with 2% CSE or control solvent for 24h. The subsequently divided groups were the same. **(D)** The proliferation of HPASMCs was measured by the EdU proliferation assay. All images are 200× magnification. Scale bar = 50 μm. **(E)** Quantitation of EdU‐positive cells. **(F)** Ki67 immunofluorescence was applied to measure the proliferation of HPASMCs. All images are 200× magnification. Scale bar = 50 μm. **(G)** Quantitation of Ki67‐positive cells. **(H)** Representative Western blot images of PCNA in HPASMCs. **(I)** Quantitation of PCNA bands based on the density. **(J)** The HPASMCs migration images. All images are 100× magnification. Scale bar = 100 μm. **(K)** Quantitation of migration cells. The data are presented as mean ± SD, n=3. *P < 0.05, **P < 0.01, ***P < 0.001.

As PH is a proliferative disease, we mainly focused on the proliferative ability of HPASMCs. We used MBD2si to discover the specific functions of the MBD2 protein in PH. Firstly, we designed three different sequences of siRNA and verified the transfection effect of the MBD2si (**Figures 3A, B**), and we found that the MBD2si1 was the most effective. As a result, we chose MBD2si1 for the subsequent experiments. Next, we performed a CCK8 assay to assess the effect of MBD2si on CSE-induced HPASMCs proliferation. As the HPASMCs proliferated due to the CSE, MBD2si prevented CSE-induced HPASMCs proliferation (**Figure 3C**). To further verify the effect of MBD2si on HPASMCs proliferation, we performed the EDU assay and found that MBD2si inhibits the multiplication of HPASMCs (**Figures 3D, E**). Simultaneously, Ki67 immunofluorescence was performed, and results similar to the EDU assay were discovered (**Figures 3F, G**). Then, we tested the PCNA protein in the HPASMCs and found that MBD2si could suppress the upregulation of PCNA due to CSE stimulation (**Figures 3H, I**). Finally, we did a transwell assay and found that MBD2si could also inhibit CSE-induced migration of the HPASMCs (**Figures 3J, K**).

### MBD2si Inhibited CSE-Induced HPASMCs Cell Cycle Transition

**Figure 4:**
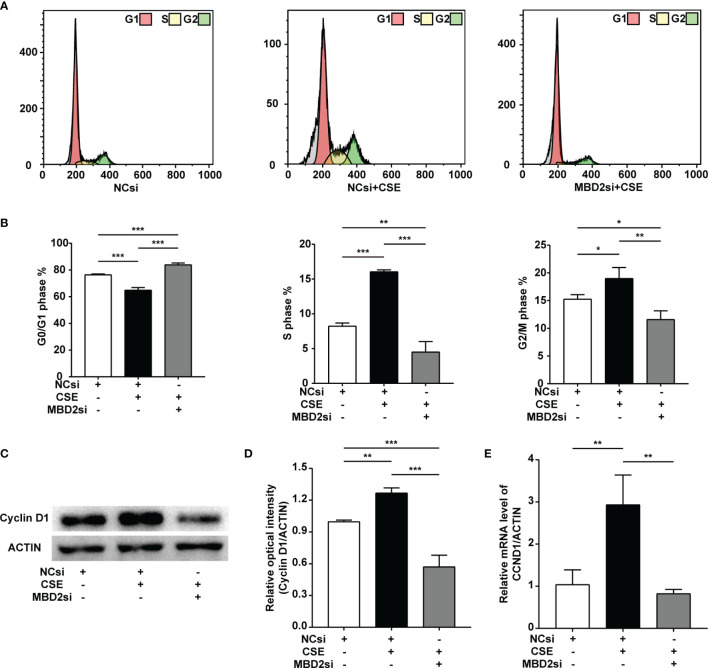
Effect of MBD2si on cell cycle progression changes in CSE-induced HPASMCs. **(A)** Representative cell cycle progression images. HPASMCs were transfected with negative control siRNA (NCsi) or MBD2si, stimulated with 2% CSE or control solvent for 24h. MBD2si changed the cell cycle progression of the HPASMCs. The subsequently divided groups were the same. **(B)** Quantitation of the cell cycle progression. **(C)** MBD2si reduced cyclin D1 protein expression in CSE-induced HPASMCs. **(D)** Quantitation of the cyclin D1 bands based on the density. **(E)** Quantitation of the CCND1 gene’s mRNA expression. The data are presented as mean ± SD, n=3. *P < 0.05, **P < 0.01, ***P < 0.001.

CSE can result in the cell cycle transition, including a decreased G0/G1 phase populations, along with increased S and G2/M phase populations. In our findings, MBD2si could suppress CSE-induced cell cycle alteration, thus raising CSE-induced G0/G1 phase populations, reducing CSE-induced S phase and G2/M phase populations (**Figures 4A, B**). According to our previous study (32–34), CSE can increase the cyclin D1 protein expression in HPASMCs. Cyclin D1 may play an effective role in the CSE-induced cell cycle transition change. Therefore, we tested the cell cycle protein, cyclin D1. The results indicated that the cyclin D1 expression in HPASMCs was elevated through CSE stimulation, while MBD2si prevented the increase in cyclin D1 protein and CCND1 mRNA (**Figures 4C-E**).

### Expression of the BMP2 Was Elevated After Treatment With MBD2si

**Figure 5:**
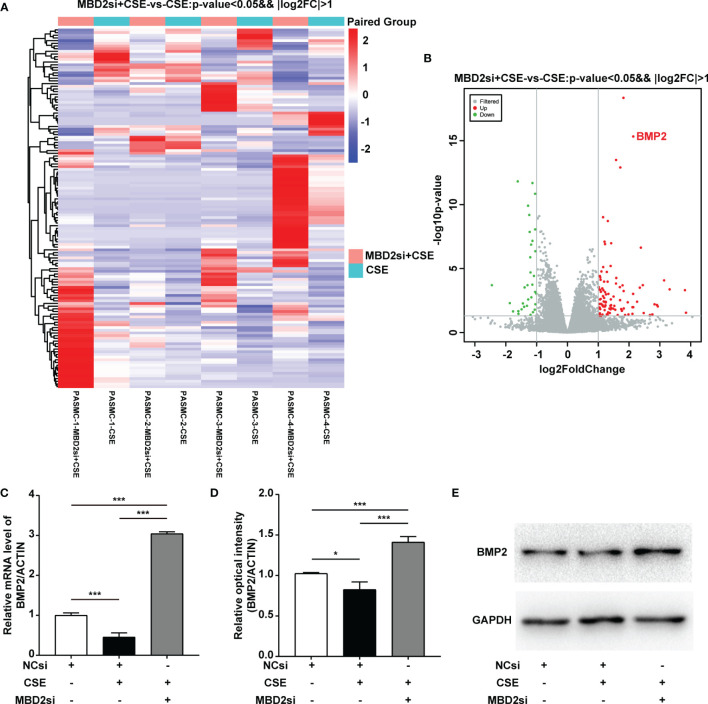
MBD2 can influence downstream BMP2 expression. **(A, B)** MBD2si induced changes in downstream genes as investigated by RNA-seq. MBD2si + CSE vs CSE, n=4, *P* < 0.05, |log2FC| > 1. **(C)** Quantitation of the BMP2 mRNA expression in HPASMCs transfected with negative control siRNA (NCsi) or MBD2si, stimulated with 2% CSE or control solvent for 24h. The subsequently divided groups are the same. **(D)** Quantitation of the BMP2 bands based on the density. **(E)** Representative Western blot images of BMP2 in HPASMCs. The data are presented as mean ± SD, n=3. *P < 0.05, ***P < 0.001.

To evaluate why MBD2si can affect the CSE-induced proliferation, migration, and cell cycle of HPASMCs, we conducted the RNA-seq assay to determine the downstream gene of MBD2. Surprisingly, we found that MBD2 was linked to the BMP2 gene (**Figure 5A, B**). Therefore, to further verify the results of the RNA-seq in the HPASMCs, we used RT-qPCR and WB assay to confirm. The results showed that CSE could lead to BMP2 downregulation, while MBD2si could increase the expression of BMP2 (**Figure 5C-E**, **Supplementary Figure 3**), which indicated that MBD2 could change the transcription of BMP2 gene and its protein expression.

### MBD2 Could Bound to BMP2’s Promoter Region and Regulated Its Expression

As a reader for interpreting DNA methylation encoded information, MBD2 tended to function by directly binding methylated CpG DNA and forming an inhibitory complex or crosstalk with the histone-modifying and chromatin-remodeling enzymes to achieve a follow-up transcription program (35).

**Figure 6:**
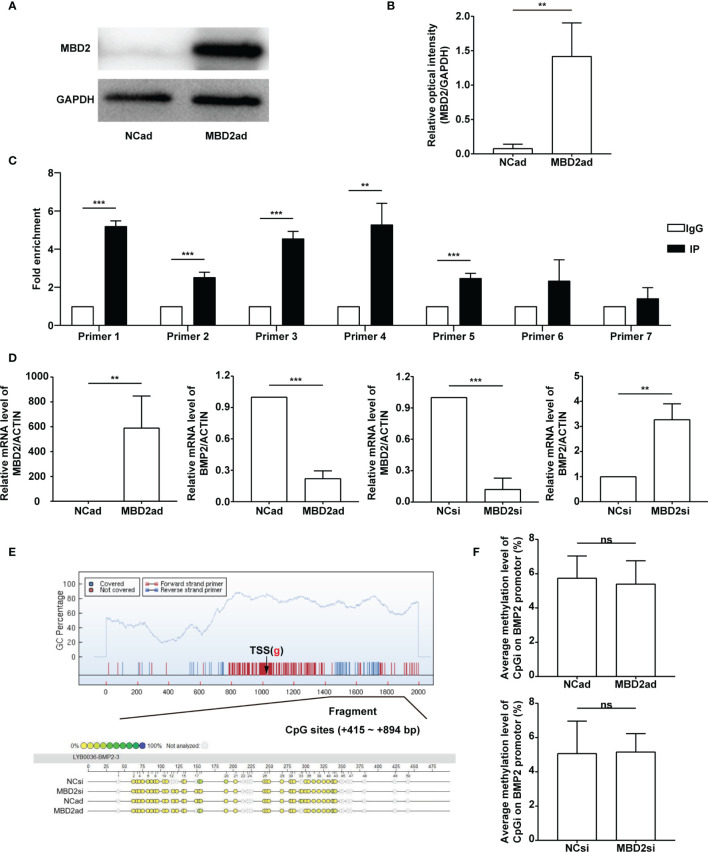
MBD2 can directly bind to the BMP2 gene’s promoter. **(A)** MBD2 overexpression adenovirus vector (MBD2ad) increases MBD2 protein expression in HPASMCs. **(B)** Quantitative analysis results of graph A based on the density of the bands. (n=3) **(C)** The ChIP-qPCR results indicate that MBD2 can directly bind to the BMP2 gene’s promoter. (n=3) **(D)** RT-qPCR results indicate that MBD2 can influence BMP2 expression at the transcriptional level. The samples were collected for the MassARRAY. (MBD2ad group, n=6; MBD2si group, n=5) **(E)** Representative images of the MassARRAY assay. **(F)** Average methylation level of CpG islands on BMP2 promoter (MBD2ad group, n=6; MBD2si group, n=5). The data are presented as mean ± SD. **P < 0.01, ***P < 0.001, ns, no significance.

To investigate the relationship between MBD2 and BMP2, we designed the overexpression adenovirus vector of MBD2 (MBD2ad, **Figures 6A, B**), and used MBD2si as previously described. We assumed MBD2 could directly bind to the promoter area of the BMP2 gene in HPASMCs. Therefore, we performed a ChIP-qPCR assay to verify our hypothesis. We designed seven primers according to the BMP2’s promoter CpG island region. The results confirmed our hypothesis by showing that MBD2 could bind to the BMP2’s promoter region (**Figure 6C**).

Futhermore, we assumed that MBD2 could influence BMP2’s methylation status. We conducted a MassARRAY assay to confirm our hypothesis. Before the MassARRAY assay, the BMP2 and the MBD2 mRNA of the samples were evaluated by RT-qPCR (**Figure 6D**), which indicated that MBD2 could directly influence BMP2’s transcription in its specific regulation mode. After the assay, we discovered that the methylation of the BMP2 gene was not changed by manipulating MBD2 (**Figures 6E, F**), indicating that MBD2 exerted its transcription repression not by regulating gene’s methylation status but by bind its promoter and gathering proteins to form a suppressive compound.

## Discussion

Our results indicate the role of increased MBD2 expression in the development of CS-induced pulmonary vascular remodeling. Herein, MBD2 were increased in the pulmonary arteries of the PH patients and CS-exposed rats. The *in vivo* findings showed that MBD2 increased along with the concentration and stimulation time of CSE. Then, it was found that the absence of MBD2 in HPASMCs could reduce the proliferation and migration caused by CSE. Furthermore, a deficiency in MBD2 could decrease the upregulation of cyclin D1 induced by CSE, and reverse the cell cycle transition after CSE stimulation. To investigate the possible mechanisms of those effects, we performed an RNA-seq assay and found that MBD2 could influence the transcription of BMP2 gene, which furtherly changed the expression of BMP2 protein. In addition, we verified with a ChIP-qPCR assay that MBD2 could bind directly to the promoter of BMP2. All in all, our research shows that MBD2 may act as a potential therapeutic target for CS-induced PH.

Of the epigenetic changes, DNA methylation is a very notable category and can profoundly affect cell function. Epigenetics is associated with CS as well as PH (11, 36). DNA methylation is featured by covalently adding a methyl group on the fifth carbon of cytosine, which forms 5-methylcytosine. This structure enables binding of the methyl binding domain proteins, followed by the recruitment of the histone-modifying and chromatin-remodeling enzymes, and leading to a chromatin-repressing structure and inhibited gene expression (37). The family of the methyl-CpG-binding domain proteins functions as a methylated reader by binding directly to the methylated CpG DNA (38). MBD2 belongs to the family of the methyl-CpG-binding domain proteins and has been connected to multiple diseases (18, 19, 39, 40), such as cancers and inflammations. As a proliferative disease comparable to tumors, PH may also be influenced by multiple tumor-associated genes, including MBD2. Other biomolecules interacted with MBD2 are broadly reported in PH relevant research. For instance, MBD2 is believed to repress the MYC gene transcription, thus suppressing glycolysis (41, 42), and MBD2 is also reported to interplay with non-coding RNA related to PH (23). Whereas in other cases, MBD2 is also associated with many proliferative diseases. For example, MBD2 is associated with cancer-related methylations in prostate cancer (43), and it is also interrelated to chronic myeloid leukemia (44). Furthermore, it is also stated that MBD2 can correlate with cancer angiogenesis (45). In most cases above, the expression of MBD2 elevated, while in some other instances, MBD2 decreased. Sometimes, MBD2 acted as a pathogenic factor, while in other cases, MBD2 served as a protective factor. MBD2 is conventionally considered a transcriptional repressor that combines inhibitory complexes (46), while in some other research, MBD2 has been regarded as a transcriptional activator that activates gene expression (47). However, it activates some regulative genes to inhibit the downstream functional genes’ transcription. Various studies have shown that methylation is multifunctional (38). Therefore, we consider its transcriptional activation may be the other manifestation of its transcriptional inhibition function. Our previous research showed that DNA methylation plays a vital part in CS-induced PH (13). Therefore, we hypothesized that MBD2 might also play a role in CS-induced PH. We conducted assays to detect the expression of MBD2 in the pulmonary arteries of the PH patients and CS-exposed rats. We also performed assays to investigate MBD2 in HPASMCs following CSE stimulation. The results indicated that MBD2 was elevated in the situations mentioned above. Our results are similar to most studies associated with MBD2 in diseases. Its elevated expression is similar to that in other proliferative diseases, suggesting that it may function as a pathogenic factor in CS-induced PH. According to the basic functions of MBD2, elevated MBD2 protein may operate by binding methylated DNA and inhibiting the transcription of the downstream functional genes. To further verify the specific effects of MBD2 in the HPASMCs, functional assays were performed.

In healthy adults, PASMCs are in their differentiated contractile state and exhibit low synthetic and proliferative activity (48). However, like other vascular smooth muscle cells (VSMCs), PASMCs are not terminally differentiated and can undergo a phenotypic transformation and change their state to a de-differentiated phenotype with a high rate of proliferation, migration, and protein synthesis in response to the environment (48, 49). CS stimulation is such an environment. Among various cellular processes, PASMCs proliferation plays a central role in the pulmonary vascular remodeling in PH (50, 51). We performed cell proliferation related assays to detect the proliferative process of the HPASMCs. We conducted CCK8 assay and EDU assay, and found that MBD2 interfering could attenuate the increased proliferation of the HPASMCs caused by CSE stimulation. The CSE stimulation can increase proliferative markers, such as PCNA and Ki67, and our results indicated that MBD2 interference could diminish those effects. Simultaneously, we tested migration and cell cycle progression functions. We found that MBD2si could decrease CSE-induced increased migration and prevent HPASMCs from cell cycle transition caused by CSE, which is consistent with the reported conclusion that MBD2 interference can attenuate migration and decelerate cycle transition (18, 52). According to our previous research (32–34, 53, 54), cyclin D1 increases in CS-induced PH, which plays a vital role. Furthermore, studies have shown that MBD2 protein knockdown can decelerate cell cycle progression (18, 52), and MBD2si can reduce the expression of cyclin D1 protein (18). Therefore, we concentrated further on cyclin D1 protein. In our study, CSE prominently increases the cyclin D1 protein and CCND1 mRNA in HPASMCs, while MBD2si can decrease those changes markedly. The ability of MBD2si to diminish cyclin D1 mechanistically explains its function to CSE-induced cell cycle transition. In conclusion, according to our findings, MBD2 interference can prominently attenuate CSE-induced HPASMCs’ proliferation, migration, and cell cycle transition. The interruption of those functions by MBD2si implies that it can attenuate CS-induced PH and serve as a therapeutic target for it. However, the underlying mechanisms remain to be further investigated.

As a member of the methyl-CpG-binding domain proteins, MBD2 can combine methylated DNA and possess the highest affinity (18, 19). Thus, we hypothesized that MBD2si could attenuate CS-induced PH by reducing MBD2’s combination to the genes that exert antiproliferative influences in HPASMCs. We performed RNA-seq to search for the downstream genes of MBD2. We compared the CSE and the MBD2si with CSE group and acquired various differential genes. After looking through multiple articles, we eventually focused on the BMP2 gene. BMP2 is part of the transforming growth factor-beta (TGF-beta) superfamily. It is extensively reported to be involved in PH as its receptor BMPR2 (1, 15, 55–58). The research has revealed that BMP2 can be originated from different cell types such as HPASMCs and endothelial cells. It can be endogenously expressed in HPASMCs, which can be synthesized and secreted to the intercellular space and can activate BMP2 receptors on adjacent HPASMCs (59). It is also reported to correlate with methylation and CS (60–62). After treatment of 5-Aza-2’-deoxycytidine (5Aza), BMP2 mRNA was reported to increase in the 5Aza treating group (61). An *in vivo* study by Andersen et al. suggested that BMP2 has a protective role in PH (63). It has also been reported that BMP2 has an antiproliferative role in PASMCs of secondary PH patients and normal subjects (59, 64). In our study, we mainly focused on the secondary (CS-induced) PH, and most literature tends to report the antiproliferative role of BMP2 in PASMCs (57–59). There is literature stating that BMP6 is down-regulated in CS-induced COPD (65). To further verify all the data and hypotheses, we performed the afterward experiments. RT-qPCR and WB were conducted to investigate whether BMP2 was actually influenced by the CSE and MBD2 to verify the results of the RNA-seq. Although there was no literature in which MBD2 is associated with BMP2, it has been stated already that MBD2 could control the BMP4 response and DNA methylation may have a role BMP4 signaling (66), while BMPR2 also seemed to be related with hypermethylation in heritable PH (15), which is all similar to our findings. Therefore, the hypothesis that MBD2 can directly bind to the BMP2 promoter seemed worthy of investigation. Hence, a ChIP-qPCR assay was performed to determine whether MBD2 could bind to BMP2 promoter, and the conclusion was that MBD2 indeed could bind to the BMP2 promoter in multiple CpG sequence sites. For verification of whether MBD2 could affect DNA methylation, MassARRAY assay was performed. The findings suggested that MBD2 didn’t directly change the methylation of BMP2 promoter. In sum, MBD2 can influence CS-induced PH by binding the BMP2 gene and downregulating its expression.

There are some limitations to our study. Although we indicate that the increased MBD2 promotes cigarette smoke-induced pulmonary vascular remodeling in *in vitro* assays, the smooth muscle cell-specific MBD2 deficient mice still should be designed to further prove the assumption. However, the MBD2 conditional knockout mice need more time to generate.

## Data Availability Statement

The datasets presented in this study can be found in online repositories. The names of the repository/repositories and accession number(s) can be found below: https://www.ncbi.nlm.nih.gov/, SRR16970307, https://www.ncbi.nlm.nih.gov/, SRR16970306, https://www.ncbi.nlm.nih.gov/, SRR16970305, https://www.ncbi.nlm.nih.gov/, SRR16970304, https://www.ncbi.nlm.nih.gov/, SRR16970303, https://www.ncbi.nlm.nih.gov/, SRR16970302, https://www.ncbi.nlm.nih.gov/, SRR16970301, https://www.ncbi.nlm.nih.gov/, SRR16970300.

## Ethics Statement

The investigations of human participants were reviewed and approved by the Human Research Ethics Committees of Tongji Hospital, Tongji Medical College, Huazhong University of Science and Technology. The participants gave their written informed consent to join the study. The animal experiments were reviewed and approved by the Animal Care and Use Committees of Tongji Hospital, Tongji Medical College, Huazhong University of Science and Technology.

## Author Contributions

JGX and JX designed the research and revised the manuscript. JW performed most of the experiments, contributed to the conception, and drafted the manuscript. QH, QL, YG, and YZ took part in cell culture experiments and analyzed and discussed the data. TW, JC, ZZ, YL, and JZ helped collect the human tissue samples and analyzed data. All authors contributed to the article and approved the submitted version.

## Funding

This study was supported by the National Natural Science Foundation of China (No. 81973986, 82170049, 82070032, 81800041), Health Research Fund of Wuhan (No. WX21Q07) and Health and family planning research project of Hubei (No. WJ2019M116).

## Conflict of Interest

The authors declare that the research was conducted in the absence of any commercial or financial relationships that could be construed as a potential conflict of interest.

## Publisher’s Note

All claims expressed in this article are solely those of the authors and do not necessarily represent those of their affiliated organizations, or those of the publisher, the editors and the reviewers. Any product that may be evaluated in this article, or claim that may be made by its manufacturer, is not guaranteed or endorsed by the publisher.

## Supplementary Material

Supplementary Figure 1 The CS-induced PH rats’ phenotypes. **(A)** Representative results of HE stain of pulmonary arteriole (outside diameter: 50-150 μm). Scale bar = 100 μm. **(B)** Quantitative analysis results of the medial wall thickness (MT%). (C) Quantitative analysis results of the right ventricular systolic pressure (RVSP). The data are presented as mean ± SD, n=3. ****P* < 0.001.

Supplementary Figure 2 MBD2 mRNA elevated after CSE stimulation. Quantitation of the MBD2 gene’s mRNA expression after 2% CSE stimulation for 24h. The data are presented as mean ± SD, n=3. ****P* < 0.001.

Supplementary Figure 3 The decrease in BMP2 protein in cigarette smoke (CS)-induced pulmonary hypertension (PH). Representative results of coimmunostaining of BMP2 and α-SMA in lung sections of the controls or PH groups, and the air group rats or smoking group rats. All images are 400× magnification. Scale bar = 20 μm.

## References

[1] Galie N Humbert M Vachiery JL Gibbs S Lang I Torbicki A. 2015 Esc/Ers Guidelines for the Diagnosis and Treatment of Pulmonary Hypertension: The Joint Task Force for the Diagnosis and Treatment of Pulmonary Hypertension of the European Society of Cardiology (Esc) and the European Respiratory Society (Ers): Endorsed By: Association for European Paediatric and Congenital Cardiology (Aepc), International Society for Heart and Lung Transplantation (Ishlt). Eur Heart J (2016) 37(1):67–119. doi: 10.1093/eurheartj/ehv317 26320113

[2] Simonneau G Gatzoulis MA Adatia I Celermajer D Denton C Ghofrani A. Updated Clinical Classification of Pulmonary Hypertension. J Am Coll Cardiol (2013) 62(25 Suppl):D34–41. doi: 10.1016/j.jacc.2013.10.029 24355639

[3] Humbert M Sitbon O Simonneau G. Treatment of Pulmonary Arterial Hypertension. N Engl J Med (2004) 351(14):1425–36. doi: 10.1056/NEJMra040291 15459304

[4] Schermuly RT Ghofrani HA Wilkins MR Grimminger F. Mechanisms of Disease: Pulmonary Arterial Hypertension. Nat Rev Cardiol (2011) 8(8):443–55. doi: 10.1038/nrcardio.2011.87 PMC7097518 21691314

[5] Montani D Chaumais MC Guignabert C Günther S Girerd B Jaïs X. Targeted Therapies in Pulmonary Arterial Hypertension. Pharmacol Ther (2014) 141(2):172–91. doi: 10.1016/j.pharmthera.2013.10.002 24134901

[6] Barberà JA Peinado VI Santos S. Pulmonary Hypertension in Chronic Obstructive Pulmonary Disease. Eur Respir J (2003) 21(5):892–905. doi: 10.1183/09031936.03.00115402 12765440

[7] Seimetz M Parajuli N Pichl A Veit F Kwapiszewska G Weisel FC. Inducible Nos Inhibition Reverses Tobacco-Smoke-Induced Emphysema and Pulmonary Hypertension in Mice. Cell (2011) 147(2):293–305. doi: 10.1016/j.cell.2011.08.035 22000010

[8] Ferrer E Peinado VI Díez M Carrasco JL Musri MM Martínez A. Effects of Cigarette Smoke on Endothelial Function of Pulmonary Arteries in the Guinea Pig. Respir Res (2009) 10(1):76. doi: 10.1186/1465-9921-10-76 19682386 PMC3224554

[9] Peinado VI Pizarro S Barbera JA. Pulmonary Vascular Involvement in Copd. Chest (2008) 134(4):808–14. doi: 10.1378/chest.08-0820 18842913

[10] Wright JL Levy RD Churg A. Pulmonary Hypertension in Chronic Obstructive Pulmonary Disease: Current Theories of Pathogenesis and Their Implications for Treatment. Thorax (2005) 60(7):605–9. doi: 10.1136/thx.2005.042994 PMC1747459 15994270

[11] Santos S Peinado VI Ramirez J Melgosa T Roca J Rodriguez-Roisin R. Characterization of Pulmonary Vascular Remodelling in Smokers and Patients With Mild Copd. Eur Respir J (2002) 19(4):632–8. doi: 10.1183/09031936.02.00245902 11998991

[12] Liu Y Zhang H Li Y Yan L Du W Wang S. Long Noncoding Rna Rps4l Mediates the Proliferation of Hypoxic Pulmonary Artery Smooth Muscle Cells. Hypertension (2020) 76(4):1124–33. doi: 10.1161/HYPERTENSIONAHA.120.14644 32772647

[13] Li Q Wu J Xu Y Liu L Xie J. Role of Rasef Hypermethylation in Cigarette Smoke-Induced Pulmonary Arterial Smooth Muscle Remodeling. Respir Res (2019) 20(1):52. doi: 10.1186/s12931-019-1014-1 30845941 PMC6407244

[14] Zhou Y Fang XL Zhang Y Feng YN Wang SS. Mir-20a-5p Promotes Pulmonary Artery Smooth Muscle Cell Proliferation and Migration by Targeting Abca1. J Biochem Mol Toxicol (2020) 34(12):e22589. doi: 10.1002/jbt.22589 32720422

[15] Liu D Yan Y Chen JW Yuan P Wang XJ Jiang R. Hypermethylation of Bmpr2 Promoter Occurs in Patients With Heritable Pulmonary Arterial Hypertension and Inhibits Bmpr2 Expression. Am J Respir Crit Care Med (2017) 196(7):925–8. doi: 10.1164/rccm.201611-2273LE 28170297

[16] Lee KW Pausova Z. Cigarette Smoking and DNA Methylation. Front Genet (2013) 4:132. doi: 10.3389/fgene.2013.00132 23882278 PMC3713237

[17] Qiu W Wan E Morrow J Cho MH Crapo JD Silverman EK. The Impact of Genetic Variation and Cigarette Smoke on DNA Methylation in Current and Former Smokers From the Copdgene Study. Epigenetics (2015) 10(11):1064–73. doi: 10.1080/15592294.2015.1106672 PMC4844199 26646902

[18] Li L Li N Liu N Huo F Zheng J. Mbd2 Correlates With a Poor Prognosis and Tumor Progression in Renal Cell Carcinoma. Onco Targets Ther (2020) 13:10001–12. doi: 10.2147/OTT.S256226 PMC7548338 33116585

[19] Wang Y Zhang L Wu GR Zhou Q Yue H Rao LZ. Mbd2 Serves as a Viable Target Against Pulmonary Fibrosis by Inhibiting Macrophage M2 Program. Sci Adv (2021) 7(1):eabb6075. doi: 10.1126/sciadv.abb6075 33277324 PMC7775789

[20] Leighton G Williams DC Jr. The Methyl-Cpg-Binding Domain 2 and 3 Proteins and Formation of the Nucleosome Remodeling and Deacetylase Complex. J Mol Biol (2019) 432(6):1624–39. doi: 10.1016/j.jmb.2019.10.007 PMC7156326 31626804

[21] Du Q Luu PL Stirzaker C Clark SJ. Methyl-Cpg-Binding Domain Proteins: Readers of the Epigenome. Epigenomics (2015) 7(6):1051–73. doi: 10.2217/epi.15.39 25927341

[22] Xie Y Wang F Yu J Zhang J Liu Y Li M. Silencing of Mbd2 and Ezh2 Inhibits the Proliferation of Colorectal Carcinoma Cells by Rescuing the Expression of Sfrp. Oncol Rep (2021) 46(6):250. doi: 10.3892/or.2021.8201 34617573 PMC8524315

[23] Yuan K Xie K Fox J Zeng H Gao H Huang C. Decreased Levels of Mir-224 and the Passenger Strand of Mir-221 Increase Mbd2, Suppressing Maspin and Promoting Colorectal Tumor Growth and Metastasis in Mice. Gastroenterology (2013) 145(4):853–64.e9. doi: 10.1053/j.gastro.2013.06.008 23770133 PMC3783518

[24] Huluka DK Mekonnen D Abebe S Meshesha A Mekonnen D Deyessa N. Prevalence and Risk Factors of Pulmonary Hypertension Among Adult Patients With Hiv Infection in Ethiopia. Pulm Circ (2020) 10(4):2045894020971518. doi: 10.1177/2045894020971518 33282203 PMC7691916

[25] Joppa P Petrasova D Stancak B Tkacova R. Systemic Inflammation in Patients With Copd and Pulmonary Hypertension. Chest (2006) 130(2):326–33. doi: 10.1378/chest.130.2.326 16899829

[26] Olsson KM Olle S Fuge J Welte T Hoeper MM Lerch C. Cxcl13 in Idiopathic Pulmonary Arterial Hypertension and Chronic Thromboembolic Pulmonary Hypertension. Respir Res (2016) 17:21. doi: 10.1186/s12931-016-0336-5 26927848 PMC4770535

[27] Rohm I Grun K Muller LM Kretzschmar D Fritzenwanger M Yilmaz A. Increased Serum Levels of Fetal Tenascin-C Variants in Patients With Pulmonary Hypertension: Novel Biomarkers Reflecting Vascular Remodeling and Right Ventricular Dysfunction? Int J Mol Sci (2017) 18(11):239–49. doi: 10.3390/ijms18112371 PMC5713340 29117120

[28] Song XW Zou LL Cui L Li SH Qin YW Zhao XX. Plasma Mir-451 With Echocardiography Serves as a Diagnostic Reference for Pulmonary Hypertension. Acta Pharmacol Sin (2018) 39(7):1208–16. doi: 10.1038/aps.2018.39 PMC6289348 29795360

[29] Wang J Wu J Zhu X Chen J Zhao J Xu Y. Absence of the Mfg-E8 Gene Prevents Hypoxia-Induced Pulmonary Hypertension in Mice. J Cell Physiol (2021) 236(1):587–600. doi: 10.1002/jcp.29885 32592231 PMC7689852

[30] Wang Z Zhao J Wang T Du X Xie J. Fine-Particulate Matter Aggravates Cigarette Smoke Extract-Induced Airway Inflammation *Via* Wnt5a-Erk Pathway in Copd. Int J Chron Obstruct Pulmon Dis (2019) 14:979–94. doi: 10.2147/COPD.S195794 PMC6512785 31190784

[31] Wang J Li Q Xie J Xu Y. Cigarette Smoke Inhibits Baff Expression and Mucosal Immunoglobulin a Responses in the Lung During Influenza Virus Infection. Respir Res (2015) 16:37. doi: 10.1186/s12931-015-0201-y 25849069 PMC4364338

[32] Wang R Xu YJ Liu XS Zeng DX Xiang M. Ccn2 Promotes Cigarette Smoke-Induced Proliferation of Rat Pulmonary Artery Smooth Muscle Cells Through Upregulating Cyclin D1 Expression. J Cell Biochem (2012) 113(1):349–59. doi: 10.1002/jcb.23361 21928352

[33] Zeng DX Xu YJ Liu XS Wang R Xiang M. Cigarette Smoke Extract Induced Rat Pulmonary Artery Smooth Muscle Cells Proliferation *Via* Pkcalpha-Mediated Cyclin D1 Expression. J Cell Biochem (2011) 112(8):2082–8. doi: 10.1002/jcb.23131 21465534

[34] Xaing M Liu X Zeng D Wang R Xu Y. Changes of Protein Kinase Calpha and Cyclin D1 Expressions in Pulmonary Arteries From Smokers With and Without Chronic Obstructive Pulmonary Disease. J Huazhong Univ Sci Technol Med Sci (2010) 30(2):159–64. doi: 10.1007/s11596-010-0205-2 20407865

[35] Yue T Sun F Wang F Yang C Luo J Rong S. Mbd2 Acts as a Repressor to Maintain the Homeostasis of the Th1 Program in Type 1 Diabetes by Regulating the Stat1-Ifn-Gamma Axis. Cell Death Differ (2021) 29(1):218-29. doi: 10.1038/s41418-021-00852-6 PMC8738722 34420035

[36] Gamen E Seeger W Pullamsetti SS. The Emerging Role of Epigenetics in Pulmonary Hypertension. Eur Respir J (2016) 48(3):903–17. doi: 10.1183/13993003.01714-2015 27492834

[37] Tsou PS Varga J O'Reilly S. Advances in Epigenetics in Systemic Sclerosis: Molecular Mechanisms and Therapeutic Potential. Nat Rev Rheumatol (2021) 17(10):596–607. doi: 10.1038/s41584-021-00683-2 34480165

[38] Zhu H Wang G Qian J. Transcription Factors as Readers and Effectors of DNA Methylation. Nat Rev Genet (2016) 17(9):551–65. doi: 10.1038/nrg.2016.83 PMC5559737 27479905

[39] Xie Y Liu B Pan J Liu J Li X Li H. Mbd2 Mediates Septic Aki Through Activation of Pkceta/P38mapk and the Erk1/2 Axis. Mol Ther Nucleic Acids (2021) 23:76–88. doi: 10.1016/j.omtn.2020.09.028 33335794 PMC7723772

[40] Liu Z Sun L Cai Y Shen S Zhang T Wang N. Hypoxia-Induced Suppression of Alternative Splicing of Mbd2 Promotes Breast Cancer Metastasis *Via* Activation of Fzd1. Cancer Res (2021) 81(5):1265–78. doi: 10.1158/0008-5472.CAN-20-2876 33402389

[41] Sun P Wang N Zhao P Wang C Li H Chen Q. Circulating Exosomes Control Cd4(+) T Cell Immunometabolic Functions *Via* the Transfer of Mir-142 as a Novel Mediator in Myocarditis. Mol Ther (2020) 28(12):2605–20. doi: 10.1016/j.ymthe.2020.08.015 PMC7704792 32882180

[42] Zhang C Ma C Zhang L Zhang L Zhang F Ma M. Mir-449a-5p Mediates Mitochondrial Dysfunction and Phenotypic Transition by Targeting Myc in Pulmonary Arterial Smooth Muscle Cells. J Mol Med (Berl) (2019) 97(3):409–22. doi: 10.1007/s00109-019-01751-7 30715622

[43] Stirzaker C Song JZ Ng W Du Q Armstrong NJ Locke WJ. Methyl-Cpg-Binding Protein Mbd2 Plays a Key Role in Maintenance and Spread of DNA Methylation at Cpg Islands and Shores in Cancer. Oncogene (2017) 36(10):1328–38. doi: 10.1038/onc.2016.297 27593931

[44] Cheng L Tang Y Chen X Zhao L Liu S Ma Y. Deletion of Mbd2 Inhibits Proliferation of Chronic Myeloid Leukaemia Blast Phase Cells. Cancer Biol Ther (2018) 19(8):676–86. doi: 10.1080/15384047.2018.1450113 PMC6067900 29565710

[45] Zhu D Hunter SB Vertino PM Van Meir EG. Overexpression of Mbd2 in Glioblastoma Maintains Epigenetic Silencing and Inhibits the Antiangiogenic Function of the Tumor Suppressor Gene Bai1. Cancer Res (2011) 71(17):5859–70. doi: 10.1158/0008-5472.CAN-11-1157 PMC3165103 21724586

[46] Ng HH Zhang Y Hendrich B Johnson CA Turner BM Erdjument-Bromage H. Mbd2 Is a Transcriptional Repressor Belonging to the Mecp1 Histone Deacetylase Complex. Nat Genet (1999) 23(1):58–61. doi: 10.1038/12659 10471499

[47] Alvarado S Wyglinski J Suderman M Andrews SA Szyf M. Methylated DNA Binding Domain Protein 2 (Mbd2) Coordinately Silences Gene Expression Through Activation of the Microrna Hsa-Mir-496 Promoter in Breast Cancer Cell Line. PLoS One (2013) 8(10):e74009. doi: 10.1371/journal.pone.0074009 24204564 PMC3812180

[48] Hu L Wang J Huang H Yu Y Ding J Yu Y. Ythdf1 Regulates Pulmonary Hypertension Through Translational Control of Maged1. Am J Respir Crit Care Med (2021) 203(9):1158–72. doi: 10.1164/rccm.202009-3419OC 33465322

[49] Tajsic T Morrell NW. Smooth Muscle Cell Hypertrophy, Proliferation, Migration and Apoptosis in Pulmonary Hypertension. Compr Physiol (2011) 1(1):295–317. doi: 10.1002/cphy.c100026 23737174

[50] Zhang J Li Y Qi J Yu X Ren H Zhao X. Circ-Calm4 Serves as an Mir-337-3p Sponge to Regulate Myo10 (Myosin 10) and Promote Pulmonary Artery Smooth Muscle Proliferation. Hypertension (2020) 75(3):668–79. doi: 10.1161/HYPERTENSIONAHA.119.13715 32008463

[51] Zhang L Zeng XX Li YM Chen SK Tang LY Wang N. Keratin 1 Attenuates Hypoxic Pulmonary Artery Hypertension by Suppressing Pulmonary Artery Media Smooth Muscle Expansion. Acta Physiol (Oxf) (2021) 231(2):e13558. doi: 10.1111/apha.13558 32920982

[52] Zhou K Zhou M Cheng L Chen X Wang X Chu Y. Loss of Mbd2 Attenuates Mll-Af9-Driven Leukemogenesis by Suppressing the Leukemic Cell Cycle *Via* Cdkn1c. Oncogenesis (2021) 10(11):79. doi: 10.1038/s41389-021-00366-3 34789717 PMC8599466

[53] Zeng DX Liu XS Xu YJ Wang R Xiang M Xiong WN. Plasmid-Based Short Hairpin Rna Against Cyclin D1 Attenuated Pulmonary Vascular Remodeling in Smoking Rats. Microvasc Res (2010) 80(1):116–22. doi: 10.1016/j.mvr.2010.03.002 20227424

[54] Xiang M Xu YJ Liu XS Zeng DX. Cigarette Smoke Extract Promotes Human Pulmonary Artery Smooth Muscle Cells Proliferation Through Protein Kinase C Alpha-Dependent Induction of Cyclin D1. Chin Med J (Engl) (2010) 123(24):3663–70. doi: 10.3760/cma.j.issn.0366-6999.2010.24.028 22166648

[55] Nakaoka T Gonda K Ogita T Otawara-Hamamoto Y Okabe F Kira Y. Inhibition of Rat Vascular Smooth Muscle Proliferation *in Vitro* and *in Vivo* by Bone Morphogenetic Protein-2. J Clin Invest (1997) 100(11):2824–32. doi: 10.1172/jci119830 PMC508488 9389748

[56] Morse JH. Bone Morphogenetic Protein Receptor 2 Mutations in Pulmonary Hypertension. Chest (2002) 121(3 Suppl):50s–3s. doi: 10.1378/chest.121.3_suppl.50s 11893684

[57] Hansmann G de Jesus Perez VA Alastalo TP Alvira CM Guignabert C Bekker JM. An Antiproliferative Bmp-2/Ppargamma/Apoe Axis in Human and Murine Smcs and Its Role in Pulmonary Hypertension. J Clin Invest (2008) 118(5):1846–57. doi: 10.1172/JCI32503 PMC2276393 18382765

[58] Calvier L Chouvarine P Legchenko E Hoffmann N Geldner J Borchert P. Ppargamma Links Bmp2 and Tgfbeta1 Pathways in Vascular Smooth Muscle Cells, Regulating Cell Proliferation and Glucose Metabolism. Cell Metab (2017) 25(5):1118–34.e7. doi: 10.1016/j.cmet.2017.03.011 28467929

[59] Zhang S Fantozzi I Tigno DD Yi ES Platoshyn O Thistlethwaite PA. Bone Morphogenetic Proteins Induce Apoptosis in Human Pulmonary Vascular Smooth Muscle Cells. Am J Physiol Lung Cell Mol Physiol (2003) 285(3):L740–54. doi: 10.1152/ajplung.00284.2002 12740218

[60] Wang Z Liang W Ma C Wang J Gao X Wei L. Macrophages Inhibit Ciliary Protein Levels by Secreting Bmp-2 Leading to Airway Epithelial Remodeling Under Cigarette Smoke Exposure. Front Mol Biosci (2021) 8:663987. doi: 10.3389/fmolb.2021.663987 33981724 PMC8107431

[61] Kobayashi F Uehara O Ito C Furusawa M Abiko Y Muramatsu T. DNA Methylation of Gja1, Bmp2 and Bmp4 in a Human Cementoblast Cell Line Induced by Lipopolysaccharide. Int Endod J (2020) 53(6):804–11. doi: 10.1111/iej.13275 32011747

[62] Fu B Wang H Wang J Barouhas I Liu W Shuboy A. Epigenetic Regulation of Bmp2 by 1,25-Dihydroxyvitamin D3 Through DNA Methylation and Histone Modification. PLoS One (2013) 8(4):e61423. doi: 10.1371/journal.pone.0061423 23620751 PMC3631216

[63] Anderson L Lowery JW Frank DB Novitskaya T Jones M Mortlock DP. Bmp2 and Bmp4 Exert Opposing Effects in Hypoxic Pulmonary Hypertension. Am J Physiol Regul Integr Comp Physiol (2010) 298(3):R833–42. doi: 10.1152/ajpregu.00534.2009 PMC2838658 20042692

[64] Fantozzi I Huang W Zhang J Zhang S Platoshyn O Remillard CV. Divergent Effects of Bmp-2 on Gene Expression in Pulmonary Artery Smooth Muscle Cells From Normal Subjects and Patients With Idiopathic Pulmonary Arterial Hypertension. Exp Lung Res (2005) 31(8):783–806. doi: 10.1080/01902140500461026 16368652 PMC1409757

[65] Verhamme FM De Smet EG Van Hooste W Delanghe J Verleden SE Joos GF. Bone Morphogenetic Protein 6 (Bmp-6) Modulates Lung Function, Pulmonary Iron Levels and Cigarette Smoke-Induced Inflammation. Mucosal Immunol (2019) 12(2):340–51. doi: 10.1038/s41385-018-0116-2 30542109

[66] Ampuja M Rantapero T Rodriguez-Martinez A Palmroth M Alarmo EL Nykter M. Integrated Rna-Seq and Dnase-Seq Analyses Identify Phenotype-Specific Bmp4 Signaling in Breast Cancer. BMC Genomics (2017) 18(1):68. doi: 10.1186/s12864-016-3428-1 28077088 PMC5225521

