# Supplementary figures and images for: Increased Methyl-CpG-Binding Domain Protein 2 Promotes Cigarette Smoke-Induced Pulmonary Hypertension
Source: Front Oncol. 2022 Jun 16;12:879793. doi: 10.3389/fonc.2022.879793 (PMC9243313; doi:10.3389/fonc.2022.879793)

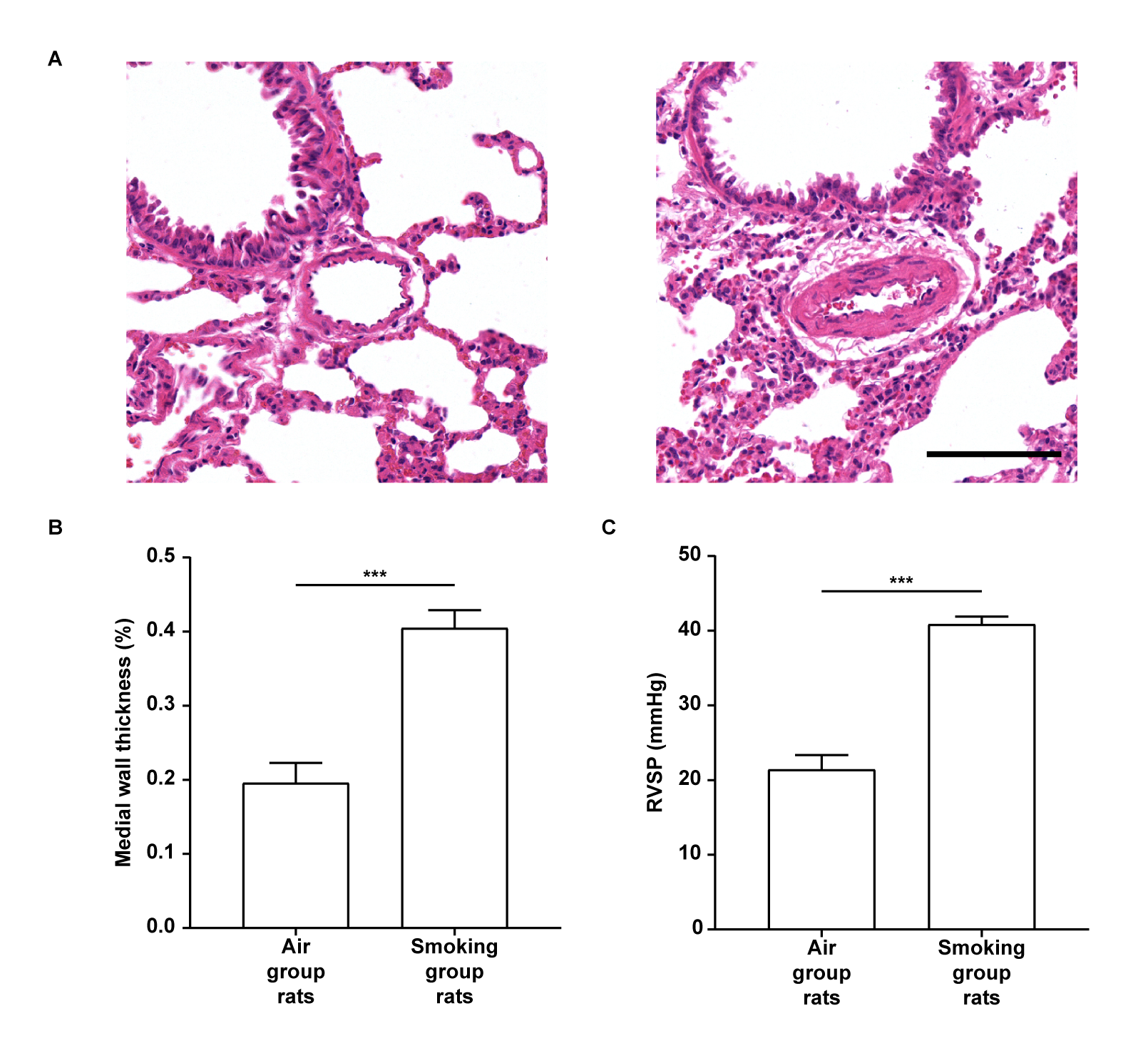

Supplement: Supplementary Figure 1 — The CS-induced PH rats’ phenotypes. (A) Representative results of HE stain of pulmonary arteriole (outside diameter: 50-150 μm). Scale bar = 100 μm. (B) Quantitative analysis results of the medial wall thickness (MT%). (C) Quantitative analysis results of the right ventricular systolic pressure (RVSP). The data are presented as mean ± SD, n=3. ***P < 0.001. [file Image_1.tif]

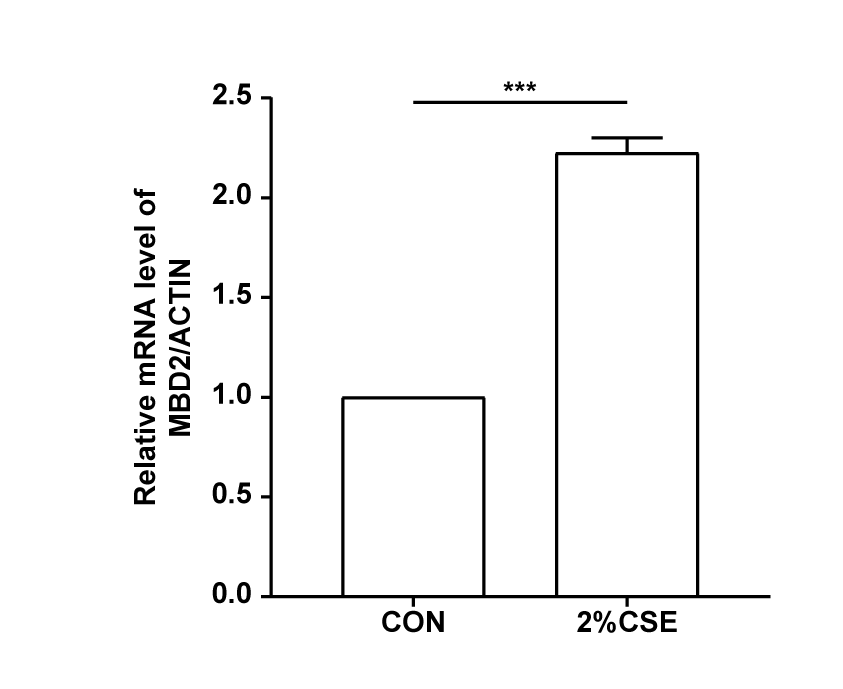

Supplement: Supplementary Figure 2 — MBD2 mRNA elevated after CSE stimulation. Quantitation of the MBD2 gene’s mRNA expression after 2% CSE stimulation for 24h. The data are presented as mean ± SD, n=3. ***P < 0.001. [file Image_2.tif]

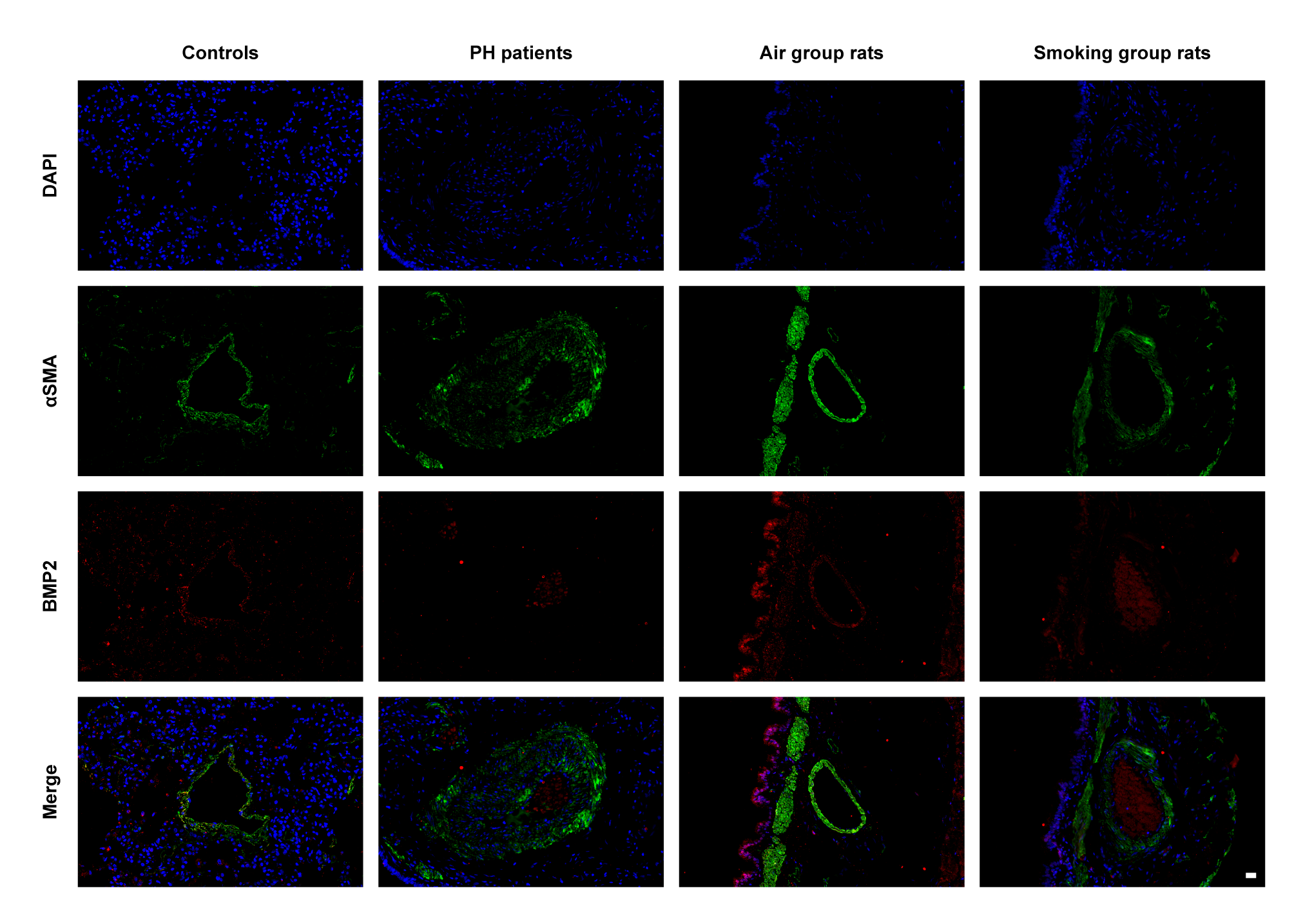

Supplement: Supplementary Figure 3 — The decrease in BMP2 protein in cigarette smoke (CS)-induced pulmonary hypertension (PH). Representative results of coimmunostaining of BMP2 and α-SMA in lung sections of the controls or PH groups, and the air group rats or smoking group rats. All images are 400× magnification. Scale bar = 20 μm. [file Image_3.tif]
